# *In vivo* effects of cadmium on signaling and secretion of pituitary gonadotrophs in male mice are time-dependent

**DOI:** 10.1530/JOE-25-0161

**Published:** 2025-10-07

**Authors:** Yorgui Santiago-Andres, Elizabeth Hernández Álvarez, Daniel Ochoa Gutierrez, Ofelia Morton Bermea, Tatiana Fiordelisio

**Affiliations:** ^1^ Laboratorio de Neuroendocrinología Comparada, Facultad de Ciencias, Universidad Nacional Autónoma de México, Ciudad Universitaria, Ciudad de México, México; ^2^ Posgrado en Ciencias Biológicas, Unidad de Posgrado, Edificio D, Primer Piso, Circuito de Posgrados, Ciudad Universitaria, Ciudad de México, México; ^3^ Laboratorio de ICP-MS, Instituto de Geofísica, Universidad Nacional Autónoma de México, Ciudad Universitaria, Ciudad de México, México; ^4^ Laboratorio de Biología Molecular y Genómica, Departamento de Biología Celular, Facultad de Ciencias, Universidad Nacional Autónoma de México, Ciudad Universitaria, Ciudad de México, México; ^5^ Laboratorio Nacional de Soluciones Biomiméticas para Diagnóstico y Terapia, Facultad de Ciencias, Universidad Nacional Autonoma de Mexico, Ciudad Universitaria, Ciudad de México, México

**Keywords:** gonadotroph, gonadotropin, endocrine disruptor, cadmium, reproduction, reproductive toxicity, GnRH, pituitary, calcium

## Abstract

Cadmium is a heavy metal found widely in the environment, originating from industrial emissions, mining activities, phosphate fertilizers, and cigarette smoke. It is an endocrine-disrupting chemical that mimics essential metals such as calcium and zinc, interfering with hormone signaling. Due to its long biological half-life, cadmium bioaccumulates in organisms, raising concerns about its long-term effects on endocrine and reproductive health. Cadmium’s reproductive toxicity is well documented, with studies highlighting its impact on gonadotropin regulation and testicular function. However, its specific effects on calcium (Ca^2+^) signaling in gonadotrophs remain poorly understood. This study aims to determine whether cadmium disrupts Ca^2+^-dependent signaling mechanisms essential for gonadotropin secretion. To address this, we used an adult male mouse model to assess pituitary cadmium accumulation, gonadotroph responsiveness to GnRH, and alterations in Ca^2+^ mobilization patterns. Our results show that cadmium exposure leads to pituitary bioaccumulation, prolonged endocrine disruption, and gonadotroph hyperplasia. Initially, gonadotroph responsiveness to GnRH declines, but over time, altered Ca^2+^ oscillation patterns and increased gonadotropin secretion emerge. A transition from normal oscillatory Ca^2+^ signaling to biphasic responses was observed, along with sustained phospholipase C-β (PLCβ) activation, suggesting persistent intracellular signaling disruptions. In addition, cadmium exposure resulted in testicular atrophy, increased apoptosis, and reduced sperm count. Testosterone levels declined, while the gonadotroph population increased, highlighting an imbalance in endocrine regulation. These findings suggest that cadmium induces reproductive toxicity through a combination of direct testicular damage and disruption of gonadotroph calcium signaling and hormone secretion, leading to testicular dysfunction that is relevant to public health.

## Introduction

Endocrine-disrupting chemicals such as cadmium have a significant impact on animal physiology by affecting the hypothalamus-pituitary system. They may mimic or block hormonal activities in target tissues or alter hormone production in endocrine glands, posing serious concerns for public and wildlife health (Vos *et al.* 2000, Patisaul & Belcher 2017, Patisaul 2021). Cadmium, in particular, has gained attention due to its widespread effects on the reproductive axis, leading to infertility and population decline in vertebrate species (Larison *et al.* 2000, Bhardwaj *et al.* 2024*a*,*b*). This heavy metal can bioaccumulate in the body for up to 40 years, with the main sources of exposure and intake including fuel combustion, batteries, leachate from landfill sites, mining residues, phosphate fertilizers, preservative pigments, stabilizers used in polyvinyl chloride (PVC) production, and smoking (Thompson & Bannigan 2008, Wu *et al.* 2017).

Studies carried out in rodent models using different exposure times and concentrations of cadmium have shown consistent morphological and physiological effects on the testes (Thompson & Bannigan 2008, Panchal *et al.* 2022, Panchal & Bhardwaj 2024). Cadmium rapidly damages the blood-testis barrier, impairing Sertoli and Leydig cell function, causing cell apoptosis, and decreasing germ cell production, spermatogenesis, sperm viability, and testosterone secretion (Saksena *et al.* 1977, Wong *et al.* 2004, Wu *et al.* 2017). At the morphological level, high concentrations of cadmium lead to hemorrhage, necrosis, and reduced testicular volume (Allanson & Deanesly 1962, Saksena *et al.* 1977). Furthermore, cadmium disrupted the gene expression of non-germinal testicular cells, which are essential for cell maintenance and proliferation, even after a single dose of cadmium administration (Wu *et al.* 2017). However, although it is known that gonadotrophs in the pituitary gland regulate the reproductive axis through the pulsatile release of LH and FSH, which requires an increase in intracellular calcium (Ca^2+^), and cadmium can mimic the effect of Ca^2+^ and disrupt the homeostasis of Ca^2+^-dependent pathways, the effects of this heavy metal and the specific mechanisms of action on gonadotrophs are poorly understood.

The secretion of LH and FSH is triggered by the gonadotropin-releasing hormone (GnRH) and requires a rapid increase in intracellular Ca^2+^ concentration ([Ca^2+^]_i_). GnRH activates Ca^2+^ mobilization in gonadotrophs through the GnRH receptor (GnRHR), which activates phospholipase C-β1 through its interaction with the α subunit of Gq/11 proteins, leading to generation of inositol-1,4,5-trisphosphate (IP_3_) and diacylglycerol. This process activates the cytoplasmic oscillator that releases Ca^2+^ from the endoplasmic reticulum through IP_3_ receptor (IP_3_R) channels (Stojilkovic *et al.* 2017). It also activates the membrane oscillator responsible for Ca^2+^ influx via voltage-gated Ca^2+^ channels, facilitating the increase of [Ca^2+^]_i_, the secretion of gonadotropins, and the expression of the *Gnrhr* and the gonadotropin subunits *Cga*, *Fshb*, and *Lhb* (Stojilkovic & Tomic 1996). Cadmium is known to block Ca^2+^ channel activity in excitable cells and reduce spontaneous Ca^2+^ activity in gonadotrophs, although it does not directly affect gonadotropin secretion *in vitro* (Hinkle *et al.* 1987, Stojilkovic *et al.* 1989, Swandulla & Armstrong 1989, Iida *et al.* 1991). However, *in vivo* studies have reported a rapid decrease in gonadotropin secretion that resulted in anovulation following cadmium administration, suggesting a direct effect on gonadotroph physiology rather than an indirect consequence of ovarian feedback (Paksy *et al.* 1989, Varga & Paksy 1991). In the long term, some studies reported that cadmium decreased LH levels in serum (Paksy *et al.* 1989, Pillai *et al.* 2003, Wu *et al.* 2017), which could be reversed with increased doses of GnRH, indicating a direct impact on the hypothalamus-pituitary system, although cadmium barely crosses the blood-brain barrier (Arvidson & Tjalve 1986, Wang & Du 2013). Nevertheless, other studies have reported either no change or an increase in gonadotropin levels, while observing modifications in their circadian secretion patterns (Wu *et al.* 2017, da Costa *et al.* 2021, Abudawood *et al.* 2021, Lafuente 2004). Moreover, as it has been observed that in other tissues cadmium disrupts the assembly of gap junctions, the function of the gonadotroph cell network may also be affected, potentially modifying cell–cell communication and hormone secretion (Thompson & Bannigan 2008).

In addition, in non-pituitary cells, cadmium disrupted calmodulin activity and Ca^2+^/calmodulin-dependent protein kinase II (CaMK-II), affecting cytoskeletal dynamics, gene expression, and promoting apoptosis (Choong *et al.* 2014). However, in the pituitary gland, cadmium increased the proliferation of lactotrophs and gonadotrophs, likely by interacting with estrogen receptors, to which it competitively binds at residues shared by Ca^2+^ (Allanson & Deanesly 1962, Gieske *et al.* 2007, Ronchetti *et al.* 2013). Furthermore, the discrepancies among the aforementioned studies have led to speculation that the effects of cadmium may vary depending on differences in species, sex, age, strain, concentration, and the duration of exposure evaluated (Henson & Chedrese 2004, Lafuente 2004).

The aim of the present study was to investigate whether cadmium disrupts Ca^2+^ signaling in gonadotrophs by interfering with the activity of the two Ca^2+^ oscillators that regulate gonadotropin secretion (Stojilkovic & Tomic 1996). Furthermore, our experimental model provides insights into the paradoxical effects of cadmium on the reproductive axis (Henson & Chedrese 2004, Thevenod 2009, Choong *et al.* 2014). To minimize prolonged structural alterations in the reproductive axis, we used a lower concentration and brief exposure to cadmium, allowing us to investigate the physiological changes in gonadotrophs. Our results suggest that the effect of Cd is due to a synergism of multiple mechanisms, with one mechanism predominating at different time points. Thus, the paradoxical effects of cadmium on gonadotropin secretion are the consequences of differential disruption of the two Ca^2+^ oscillators, with heterogeneous effects on reproduction.

## Materials and methods

### Animals and cadmium treatment

Animal procedures were conducted in accordance with the policies of the Mexican Official Regulation for the Care and Use of Laboratory Animals (SAGARPA NOM-062-ZOO-1999), and the protocols were approved by the Institutional Animal Care and Use Committee of the Faculty of Sciences, UNAM (protocol number CEARC PI_2019_02022). Experiments were carried out using 2-month-old male BALB/c mice, housed in a 12 h light:12 h darkness cycle with free access to standard rodent chow and water.

Cadmium treatment and experimental time points were based on previously published methods (Wu *et al.* 2017). Briefly, mice received a single intraperitoneal (i.p.) injection of cadmium chloride (1.0 mg/kg; C-2544, MERCK) dissolved in normal saline. Mice in the control group received an equivalent i.p. injection of normal saline. For all experiments, studies were conducted at 21, 35, and 56 days after cadmium injection (referred to as the control, Cd21, Cd35, and Cd56 groups, respectively).

### Determination of cadmium in blood and tissue samples

Animals from the control, Cd21, and Cd56 groups were deeply anesthetized with sodium thiopental (50 mg/kg i.p.; Laboratorios PiSA, Mexico), and blood was collected directly from the heart. Serum was obtained by allowing the blood to settle for 30 min and then centrifuging at 1,000 ***g*** for 10 min. The testes (including epididymis), pituitary, and brain (with the cortex and hypothalamus dissected and isolated) were collected, weighed, and stored at −80°C until further analysis. Samples were wet-digested with 3 mL of ultrapure concentrated nitric acid and subjected to a program in the microwave oven-assisted digestion unit, consisting of three stages for 10 min each, at temperatures of 20, 60, and 220°C (UltraWAVE, Milestone, Sorisole, Italy). Cadmium concentrations were measured in triplicate by inductively coupled plasma mass spectrometry (iCAP Qc, Thermo Scientific, Germany). For quality control, standard reference material DOLT-4 dogfish liver (National Research Council Canada, Canada) and blank samples were also analyzed. An 84.4% recovery rate was obtained for the DOLT-4 standard. Because cadmium detection by mass spectrometry requires a minimum tissue mass for accurate quantification, tissues from multiple animals were pooled. This was especially necessary for the pituitary, which weighs no more than 2 mg. Quantification was restricted to the control, Cd21, and Cd56 mice. The number of animals per pool and the total number of pools analyzed per condition are specified in the Results section.

### Immunofluorescence and tissue processing in pituitary

Animals were deeply anesthetized by injection of pentobarbital (50 mg/kg i.p.; Laboratorios PiSA, Mexico), and tissues were fixed by intracardiac perfusion with 4% paraformaldehyde in PBS. Pituitaries were extracted and post-fixed by immersion using 4% paraformaldehyde overnight. The glands were embedded in 3% low-melting-point agar (30391-023, Invitrogen, USA), and thin (30 μm) slices of the ventral pituitary were obtained using a vibrating microtome (Leica VT1000S, Leica Microsystems, USA). For immunostaining, free-floating sections were blocked at room temperature (RT) for 45 min in PBS containing 2% bovine serum (160069, MP Biomedicals, USA) and 1% Triton X-100 (93443, MERCK, USA). Primary antibodies were diluted in PBS solution containing 0.3% Triton X-100 and 2% BSA, and the sections were incubated for 48 h at 5°C. Primary antibodies were directed against FSHB, LHB, or Phospho-PLCβ, followed by application of the corresponding secondary antibodies diluted in PBS, and incubated for 2 h at RT (see Table 1 for concentration, manufacturer, and host species). DAPI (4′,6-diamidino-2-phenylindole; D1306, Invitrogen) was used for counterstaining, and sections were mounted using Mowiol.

**Table 1:** List of antibodies used in this study.

Target	Antibody name	Host species	Dilution	Manufacturer	Research source identifier
FSHB	Rabbit anti-rat FSH antiserum	Rabbit	1:250	A.F. Parlow National Hormone and Peptide Program	AB_2687903
LHB	Rabbit anti-rat LHβ	Rabbit	1:250	A.F. Parlow National Hormone and Peptide Program	AB_2665511
LHB	Anti-bovine LH antibody	Mouse	1:250	Dr Janet Roser, Department of Animal Science, University of California, Davis	AB_2665514
Cleaved caspase-3	Cleaved Caspase-3 (Asp175) (5A1E) Rabbit mAb	Rabbit	1:250	Cell Signaling Technology	AB_2070042
Phospho-PLCβ3 (Ser1105)	Phospho-PLCβ3 (Ser1105) Polyclonal	Rabbit	1:250	Thermo Fisher Scientific	AB_2554692
Rabbit IgG (H+L)	Alexa Fluor 488 AffiniPure F(ab′) Fragment Donkey Anti-Rabbit IgG (H+L)	Donkey	1:150	Jackson ImmunoResearch Labs	AB_2340619
Mouse IgG (H+L)	Alexa Fluor 647-AffiniPure F(ab′)2 Fragment Donkey Anti-Mouse IgG	Donkey	1:150	Jackson ImmunoResearch Labs	AB_2340865

Images were captured using a Leica TCS SP8 confocal microscope (Leica Microsystems, Germany). Similar image parameters (laser power, gain, pinhole, and wavelengths) were maintained for each slice. Whole-slice reconstructions were generated using a 20× objective, and Z-stacks were acquired individually for each channel. Subsequently, specific areas were selected and imaged using a 63× oil-immersion objective. For image analysis and processing, the reconstructions were collapsed to generate maximum intensity projections, and cells were identified and manually counted using ImageJ (Image Analysis in Java, NIH, USA).

### Real-time quantitative PCR

For gene expression analysis, total RNA was isolated from groups of two mice per sample using the TRIzol method (10296028, AMBION by Life Technologies, USA) and quantified using a BioTek Cytation 5 system (Agilent Technologies, USA). Then, cDNA generation and SYBR green-based real-time quantitative PCR (RT-qPCR) assays were performed with a 1:20 RNA dilution using the one-step SCRIPT RT-qPCR ProbesMaster kit (PCR 512S, Jena Bioscience, Germany) and a Rotor-Gene Q system (QIAGEN, Germany). All RT-qPCR assays were performed in duplicate. The results of RNA quantification were analyzed using the comparative 2^−ΔΔCt^ method, which enabled comparison of relative expression levels between different physiological conditions (Livak & Schmittgen 2001). Data were normalized to the geometric mean expression of *Rpl19*, used as the housekeeping gene (see primer list in Table 2).

**Table 2:** Primers used for real-time quantitative PCR.

Gene name	Primer
*Gnrhr* (F)	TGA TTA GCC TGG ACC GCT CCC T
*Gnrhr* (R)	GAA GGC CTG ATG CCA CCA CTG T
*Lhb* (F)	ACC TTC ACC ACC AGC ATC TGT GC
*Lhb* (R)	GGA GGT CAC AGG CCA TTG GTT GA
*Fshb* (F)	TTG GTG TGC GGG CTA CTG CT
*Fshb* (R)	TGG GCG AAC GGC AAT GTC CA
*Cga* (F)	CGA CAA TCA CCT GCC CAG AAC ACA
*Cga* (R)	CCC TGG AGA AGC AAC AGC CCA T
*Rpl19* (F)	TGC CAA TGC TCG GAT GCC TGA
*Rpl19* (R)	GCG CTT TCG TGC TTC CTT GGT

### Perifusion system for calcium imaging

The perifusion system was calibrated using 0.5 μg/mL of fluorescein sodium salt (F6377, MERCK) in deionized water, a concentration corresponding to the mean fluorescence values observed in *ex vivo* pituitary cells loaded with the intracellular calcium indicator Fluo 4-AM (F14202, Invitrogen). Deionized water was perfused for 1 min, followed by 30 s of fluorescein, and then washed out with deionized water for the remainder of the recording. Fluorescence intensity curves were generated to analyze the kinetics of fluorescence rise and decay, optimizing both the time required to reach peak intensity following fluorescein administration and the time needed for fluorescence to return to baseline levels. In addition, we ensured sustained maximal fluorescence intensity during the fluorescein perfusion phase.

Image acquisition of fluorescence intensity changes was performed using an epifluorescence stereomicroscope (M205FA, objective 0.35 NA, PlanAPO 2×, Leica Microsystems, Germany). Images were captured by a 14-bit, 512 × 512 pixel back-illuminated CoolSNAP HQ2 CCD camera (HyQ, Roper Scientific, USA) every 200 ms using a 488 nm bandpass filter for excitation and a 510 nm filter for emission with μManager software (Open Imaging, NIH, USA).

### Calcium signal measurements

For intracellular Ca^2+^ imaging, animals were deeply anesthetized by injection of pentobarbital (50 mg/kg i.p.; Laboratorios PiSA, Mexico), decapitated, and the pituitaries were immediately dissected in Ringer’s solution (130 mM NaCl, 3 mM KCl, 0.5 mM NaH_2_PO_4_, 2 mM MgCl_2_, 2 mM CaCl_2_, 1 mM NaHCO_3_, 5 mM HEPES, and 5 mM glucose; pH 7.4) bubbled with 95% O_2_ and 5% CO_2_. Whole pituitaries were incubated for 30 min in Fluo 4-AM (20 μM), diluted in a mixture of DMSO (0.01%, wt/vol; D2650, MERCK) and pluronic acid (0.005%, wt/vol; P6867, MERCK) in Ringer’s solution (37°C, 95% O_2_, and 5% CO_2_). The gland was then quickly mounted on a perfusion chamber using 3% low-melting-point agar (A1296, MERCK) to prevent movement during continuous perfusion with Ringer’s solution (2 mL/min) throughout image acquisition. All Ca^2+^ recordings were obtained from the ventral side of the pituitary using an epifluorescence stereomicroscope, with the same parameters mentioned previously. In all Ca^2+^ recordings, a total of 1,000 images were captured, and pharmacological treatments were applied by perfusing the cells with the corresponding stimulus dissolved in Ringer’s solution. For each pituitary preparation, basal Ca^2+^ activity was monitored before any stimulus application, and a final high-potassium stimulus (140 mM KCl, 10 mM HEPES, and 2 mM CaCl_2_; pH 7.4) was applied for 30 s at the end of the experiments to assess cell viability.

Gonadotroph patterns of cytosolic Ca^2+^ increase in response to GnRH stimulation (10 nM, 30 s; H-4005.0025, BACHEM AG) were analyzed in the control, Cd21, Cd35, and Cd56 groups as previously described (Toufaily *et al.* 2021). The role of L-type Ca^2+^ channels in Ca^2+^ dynamics – the main pathway for extracellular Ca^2+^ entry in gonadotrophs – was evaluated using the L-type Ca^2+^ channel blocker nifedipine (20 µM, 30 s; N-120, Alomone Labs), as described previously (Stojilkovic 2006). In addition, we tested whether the effect of cadmium on gonadotroph Ca^2+^ mobilization was due to blocking voltage-gated Ca^2+^ channels at the membrane level or via intracellular signaling mechanisms. To this end, pituitaries were perifused with Ca^2+^-free Ringer’s solution (138 mM NaCl, 2.5 mM KCl, 1.25 mM NaH_2_PO_4_, 3 mM NaHCO_3_, 10 mM HEPES, 11 mM glucose, 5 mM MgCl_2_, and 5 mM EGTA; pH 7.4 and bubbled with 95% O_2_ and 5% CO_2_), and then cells were stimulated with GnRH and a high-potassium solution (with or without Ca^2+^) for 30 s.

Image processing was performed using FIJI software (Image Analysis in Java, NIH, USA) to manually select regions of interest (ROIs) and Igor Pro software (WaveMetrics Inc.) to correct the time-series slope for photobleaching and to obtain normalized values of fluorescence intensity based on the formula ΔF/F = F/Fmin. ROI selection was based on cells that exhibited an increase in Ca^2+^ concentration during the high-potassium stimulus, and these cells represent 100% of the analyzed population in each experiment. The presence/absence of basal Ca^2+^ activity (defined as Ca^2+^ transients greater than 10% of Fmin within a 4-min epoch), the number of oscillations, amplitude, and area under the curve were quantified to assess changes in Ca^2+^ mobilization.

### Histological staining and immunofluorescence in testes

After tissue fixation with PFA as described above, the testes were immersed in Bouin’s solution (HT10132, MERCK) overnight and then stored in 70% ethanol until further processing. The paraffin-embedded tissues were sectioned into 7-μm-thick slices, stained with hematoxylin-eosin according to standard procedures, and imaged.

For antigen retrieval of PFA-fixed testis tissue, 10-μm-thick sections were pretreated with 10 mM sodium citrate for 10 min. For immunofluorescence analysis, sections were blocked at RT for 45 min in PBS containing 1% Triton X-100 and 2% bovine serum. The sections were then incubated with a caspase-3 antibody diluted in PBS containing 2% bovine serum and 0.1% Triton X-100 at 4°C for 48 h. Subsequently, an appropriate secondary antibody was applied for 2 h at RT (see Table 1). Finally, samples were counterstained with DAPI and mounted using Mowiol. All images were captured using a confocal microscope (Leica TCS SP8, Leica Microsystems, Germany) equipped with a 20× objective.

### Sperm count

Testes from animals used for real-time qPCR (as previously described) were dissected and weighed, and the caudal epididymis was isolated for sperm quantification. The caudal epididymis was placed in 1 mL of Ringer’s solution and minced into small fragments using a surgical blade. To ensure effective sperm dispersion from the tissue, samples were incubated in a 95% O_2_ and 5% CO_2_ atmosphere for 30 min at 37°C. After incubation, cells were automatically quantified using a CASY TT counter system (Roche Innovatis, Germany) with a dilution factor of 10 μL of sperm suspension in 10 mL of CASYton, using two independent measurements per sample, and following the manufacturer’s instructions.

### Hormone quantification in blood samples

Blood samples from animals used for calcium imaging were collected at the time of decapitation, as previously described. Whole blood samples were allowed to settle for 30 min and then centrifuged at 1,000 ***g*** for 10 min. Serum was separated and stored at −80°C until hormone quantification. Pituitary hormones (LH and FSH), testosterone, and estradiol levels were quantified using an immunobead-based Milliplex magnetic assay, following the manufacturer’s recommendations (MPTMAG-49 K and MSHMAG-21 K, Millipore Corporation, MERCK, Germany).

### Data analysis

Data sets were analyzed and plotted in R (http://www.r-project.org) using the dplyr, tidyr, pracma, rstatix, ggplot2, viridis, and pheatmap packages. Differences between groups were analyzed using the Wilcoxon signed-rank test with continuity correction. Alternatively, the Kruskal-Wallis test followed by Dunn’s multiple comparison tests was used to compare distributions across multiple groups. Group sizes were determined in accordance with ethical considerations while ensuring statistical power to obtain meaningful results, according to previous findings from our research group and the relevant literature. Results are presented as mean ± SD, with *P* < 0.05 taken as statistically significant.

## Results

### Cadmium bioaccumulation in the reproductive axis

We used plasma mass spectrometry to quantify cadmium levels in the pituitary gland and other tissues associated with the reproductive axis in control, Cd21, and Cd56 groups. For pituitaries and brain tissues, biological samples were pooled to minimize the number of animals used while ensuring high detection accuracy (see Table 3). In control mice, cadmium concentrations were either minimal or below the detection limit (0.0017 μg/L) across all analyzed tissues. In contrast, in animals of the Cd21 and Cd56 groups, the pituitary exhibited markedly elevated cadmium concentrations, followed by the testes, brain tissues, and blood (Table 3). Furthermore, maximum concentrations of cadmium were detected 21 days post-injection in all examined samples, which then declined at day 56 to less than half of the concentrations observed at day 21. Notably, the pituitary gland did not show the same rate of cadmium clearance observed in other tissues, indicating that bioaccumulation and excretion rates of cadmium are tissue-specific. These results demonstrated that a single cadmium injection (1.0 mg/kg) is bioaccumulated for at least 56 days after administration, with potential time-dependent effects on the physiology of the pituitary gland.

**Table 3:** Values of cadmium bioaccumulation among different tissues.

Tissue	Number of analyzed replicates per condition*	Cd concentration (μg/kg)			*P* value^†^
		Control	Cd21	Cd56	
Pituitary	3	22.46 ± 20.53	996.23 ± 293.52	765.26 ± 192.49	0.0250
Brain cortex	5	0.18 ± 0.10	60.25 ± 25.74	20.61 ± 6.11	0.0007
Hypothalamus	5	0.37 ± 0.18	69.55 ± 27.46	28.28 ± 10.62	0.0004
Testes	5	0.58 ± 0.38	954.36 ± 518.61	120.05 ± 31.40	0.0013
Serum	14	0.35 ± 0.33	16.88 ± 13.22	4.58 ± 2.0263	<0.0001

* Number of biological samples per replicate: five pituitaries, three brain cortices, three hypothalami, and three testes. Blood samples were analyzed individually.

^†^ Kruskal–Wallis test with Dunn’s multiple comparison and Holm correction for *P*-value adjustment.

### Reproductive consequences of cadmium

**Figure 1:**
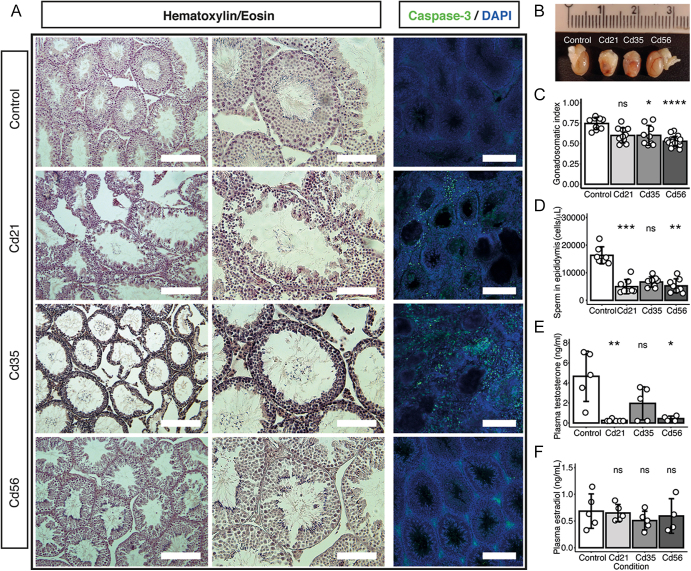
Effects of cadmium on testicular morphology and function. (A) Representative images of H&E staining on the testes showing structural modifications in the seminiferous tubules. Scale bars: 100 µm. Immunofluorescence analysis of apoptosis in testis sections using an antibody against caspase-3 (green); nuclei were counterstained with DAPI (blue). Scale bars: 200 µm. (B) Cadmium caused hemorrhage in the testes (scale bar shown in cm). (C) Testis weight was significantly reduced after cadmium injection (*P = 0.035, ****P < 0.001; *n* = 9 for control, 11 for Cd21, 9 for Cd35, and 18 for Cd56). (D) Quantification of sperm cell number in the caudal epididymis (**P = 0.001, ***P = 0.001; *n* = 7 for control, and 8 for all other groups). (E and F) Measurement of serum samples by ELISA showed a decrease in testosterone levels, with no significant differences in estradiol concentrations (*P = 0.035, **P = 0.004; for testosterone ELISA: *n* = 5 for control, 6 for Cd21, 5 for Cd35, and 4 for Cd56; for estradiol ELISA: *n* = 4 for Cd56, and 5 for all other groups). Dots represent individual mice per condition. Kruskal–Wallis test with Dunn’s multiple comparisons and Holm correction for *P*-value adjustment; ns, non-significant.

We first explored the effect of cadmium at the gonadal level. Histological analysis of the testes revealed structural alterations in the seminiferous tubules after cadmium exposure, including a loss of normal size and spherical morphology, as well as a marked reduction in the number of germ cells within the seminiferous epithelium (Fig. 1A). Then, using immunofluorescence of the caspase-3 biomarker, we observed an increase in apoptotic cells, particularly at 21 and 35 days post-cadmium injection (Fig. 1A). In addition, vascular damage was evident, with multiple hematomas observed on the surface of the testes, particularly in the Cd21 and Cd35 groups (Fig. 1B). The apparent reduction in gonadal cell content was further corroborated by measurements of the gonadosomatic index and quantification of sperm cells in the caudal epididymis (Fig. 1C and D). Notably, body mass did not change significantly after cadmium administration (data not shown). Furthermore, testicular damage was associated with a significant decrease in plasma testosterone levels, while estradiol concentrations remained unchanged (Fig. 1E and F). These findings recapitulated the testicular effects of cadmium reported in previous studies and demonstrate that a single cadmium injection modified the regulation of testicular function.

### Modifications in number and size of LH- and FSH-containing gonadotrophs

**Figure 2:**
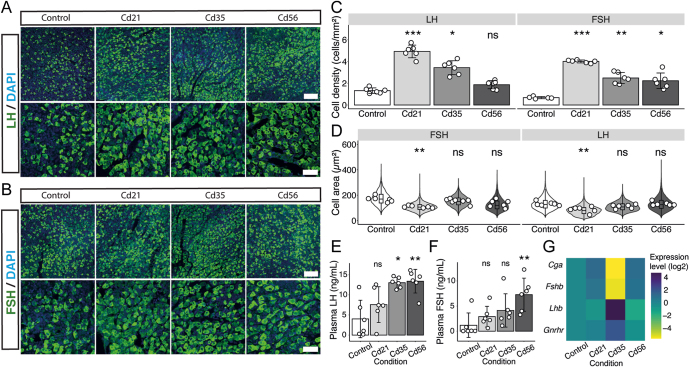
Cadmium modifies gonadotroph proliferation and gonadotropin synthesis and secretion. (A and B) Distribution of LHB- or FSHB-positive gonadotrophs (green) and nuclei (blue) in the ventral portion of the pituitary gland. Scale bars: 100 µm (top panels) and 50 µm (bottom panels). (C) Increased density of LHB- or FSHB-positive gonadotrophs following cadmium injection. Dots represent the number of animals analyzed (*n* = 6 for all groups, except for FSH in the control group where *n* = 5; *P = 0.028, **P = 0.001, ***P < 0.001). (D) Violin and box plots show heterogeneity in cell size of individual LHB-positive gonadotrophs in the control (3,430 ± 1,551 cells), Cd21 (9,215 ± 4,729 cells), Cd35 (4,133 ± 605 cells), and Cd56 (3,434 ± 352 cells) groups, and FSHB-positive gonadotrophs in control (1,387 ± 486 cells), Cd21 (6,930 ± 2,315 cells), Cd35 (5,497 ± 2,074 cells), and Cd56 (4,384 ± 1,522 cells) groups. Dots represent the mean cell area per animal. Statistical analysis was performed on these mean values (*n* = 6, except for FSH in the Cd35 group where *n* = 7; **P = 0.007). (E) LH concentration in blood samples from individual mice, represented as dots (*P = 0.035, **P = 0.006; *n* = 6). (F) FSH concentrations in blood samples from individual mice, represented as dots (**P = 0.005; *n* = 6). (G) Heatmap showing changes in gene expression of gonadotropin subunits and the GnRH receptor (four biological replicates per group). Kruskal–Wallis test with Dunn’s multiple comparisons and Holm correction for *P*-value adjustment; ns, non-significant.

To address whether cadmium bioaccumulation in the pituitary contributes to gonadotroph hyperplasia and hypertrophy, we quantified the number and size of cells containing LH and FSH via immunohistochemistry on the ventral side of the pituitary gland. In the control group, gonadotrophs were scattered throughout the tissue, but they were more densely localized in the ventral mediolateral region of the gland. The presence of FSH and LH granules gave these cells a rounded morphology, with few cytonemes making contact with adjacent cells, as shown in Fig. 2A and B and previously reported (Budry *et al.* 2011). Although approximately 90% of gonadotrophs co-express both LH and FSH (Ramaswamy & Weinbauer 2014), we found that the density of LH- and FSH-expressing cells was 1.32 ± 0.250/mm^2^ and 0.679 ± 0.099/mm^2^, respectively, indicating that there were approximately twice the number of cells containing LH in the ventral side of the gland compared to FSH cells (Fig. 2C). Furthermore, the size of FSH-expressing gonadotrophs was considerably larger (175 ± 51.6 μm^2^) than that of LH-expressing gonadotrophs (132 ± 45.5 μm^2^), likely reflecting differences in gonadotropin distribution within the cytoplasm and overall hormone content (see Fig. 2D).

We found that the number of gonadotrophs, measured as cells per area, was markedly altered following cadmium injection. The density of LH-expressing cells in the Cd21, Cd35, and Cd56 groups increased to 4.92 ± 0.582, 3.43 ± 0.638, and 1.87 ± 0.373 cells/mm^2^, respectively. Similarly, FSH-expressing cells showed a comparable pattern, reaching values of 4.00 ± 0.135, 2.49 ± 0.508, and 2.23 ± 0.714 cells/mm^2^ in the Cd21, Cd35, and Cd56 groups, respectively (Fig. 2C). However, significant differences in cell size were observed only at Cd21 in LH-expressing gonadotrophs (80.1 ± 34.1, 110 ± 33.3, and 132 ± 49.4 μm^2^ for Cd21, Cd35, and Cd56, respectively) and in FSH-expressing gonadotrophs (108.0 ± 30.2, 152 ± 39.2, and 127 ± 47.2 μm^2^ for Cd21, Cd35, and Cd56, respectively) (see Fig. 2D).

### Changes in gonadotropin secretion and gene expression

As we observed gonadotroph hyperplasia via immunohistochemistry after cadmium injection, we sought to investigate whether these cellular changes were associated with increased gonadotropin secretion. For this purpose, serum concentrations of LH and FSH were measured using ELISA. In control mice, the mean LH concentration detected was 4.66 ± 4.01 ng/mL, with no significant change observed in the Cd21 group (7.57 ± 4.45 ng/mL). However, the LH levels increased significantly in Cd35 and Cd56 mice, reaching 13.00 ± 1.32 ng/mL and 13.40 ± 2.39 ng/mL, respectively (Fig. 2E). In addition, we found a similar pattern for FSH, with elevated levels detected at 35 and 56 days post-injection, but not at 21 days. Specifically, FSH concentrations were 1.18 ± 2.44 ng/mL in control animals, 2.90 ± 2.06 ng/mL in Cd21, 4.13 ± 3.31 ng/mL in Cd35, and 7.26 ± 3.30 ng/mL in Cd56 (Fig. 2F). Consistent with the observed gonadotroph expansion, we also observed increased secretion of FSH and LH following cadmium administration, suggesting that the cellular plasticity of gonadotrophs was activated to enhance gonadotropin production. These results suggest a direct disruption of gonadotroph function in response to cadmium exposure.

To further investigate the molecular basis of gonadotroph changes, we performed RT-qPCR on pituitary RNA samples. Cadmium exposure elicited divergent expression patterns in the gonadotropin subunit genes *Lhb* and *Fshb*. Specifically, *Lhb* expression was moderately reduced in the Cd21 and Cd56 groups but showed a four-fold increase in Cd35 animals compared to control. In contrast, *Fshb* mRNA levels were elevated on days 21 and 56 but exhibited a marked decrease in the Cd35 group (Fig. 2G). These fluctuations may partially account for increased FSH and LH content and secretion observed in serum analysis.

Given that LH- and FSH-containing cells duplicate their number following cadmium exposure, we expected a more robust increase in *Lhb* and *Fshb* mRNA levels. However, we observed elevated *Fshb* expression only in Cd21 and Cd56 groups, while *Lhb* was upregulated solely in Cd35. Notably, the biological activity of LH at this point may be constrained by concurrent downregulation of *Cga*. In addition, the increase in LH-expressing cells despite reduced *Lhb* expression suggests that cadmium alters gonadotroph signaling and secretion dynamics beyond the level of transcriptional regulation. Supporting this interpretation, we also observed increased expression of the *Gnrhr*, which encodes the key hypothalamic regulator of gonadotroph physiology, GnRH (Fig. 2G).

### Intracellular calcium response to GnRH in gonadotrophs

To assess the impact of cadmium exposure on gonadotroph function, we analyzed intracellular Ca^2+^ dynamics in response to GnRH stimulation. Given that Ca^2+^ mobilization is essential for gonadotropin secretion, we examined whether cadmium affected the proportion of GnRH-responsive cells, their basal intracellular Ca^2+^ activity, or the dominant [Ca^2+^]_i_ response pattern.

Previous studies have reported that GnRH activates three distinct intracellular Ca^2+^ mobilization patterns in gonadotrophs – subumbral, oscillatory, and biphasic – associated with increasing GnRH concentrations. Low GnRH concentrations typically generate subumbral responses, whereas higher concentrations generate biphasic patterns (Iida *et al.* 1991). However, other studies have demonstrated that all three Ca^2+^ mobilization patterns can be triggered using a single GnRH concentration (Durán-Pastén & Fiordelisio 2013). For this reason, to ensure that our perifusion system delivered a stable and controlled GnRH stimulus, we calibrated it using fluorescein, which produced a square pattern of rapid increase and decline in fluorescence intensity, closely mimicking the measurements of the Fluo-4-AM Ca^2+^ sensor (see Supplementary Figure 1 (see section on Supplementary materials given at the end of the article)). This procedure allowed us to validate that the [Ca^2+^]_i_ patterns observed in our pituitary preparations were not artifacts resulting from fluctuations in GnRH concentrations but intrinsic properties of gonadotroph cells.

**Figure 3:**
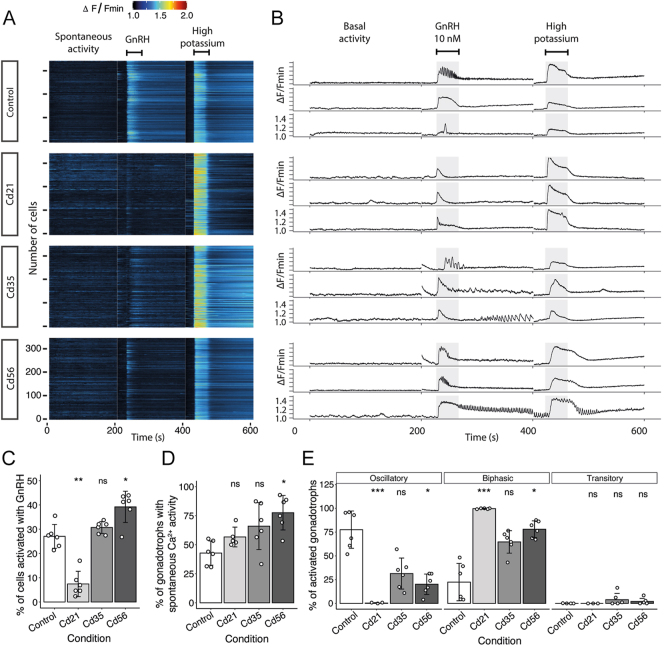
Intracellular Ca^2+^ mobilization in gonadotrophs is disrupted by cadmium. (A) Representative raster plots of pituitary cells loaded with Fluo-4 AM, showing changes in fluorescence intensity over time corresponding to variation in [Ca^2+^]_i_. Cells exhibit more spontaneous Ca^2+^ spikes and respond to high-potassium stimulus, but show a reduced response to GnRH in cadmium-treated groups. (B) Individual cells extracted from the raster plots in (A) that responded to GnRH and were classified as gonadotrophs in this study. Three main Ca^2+^ mobilization patterns can be observed in the control group – oscillatory (top), biphasic (middle), and transitory (bottom) – with disrupted patterns following cadmium injection. Gray bars indicate the 30 s of GnRH and high-potassium application. (C) Relative to the number of cells responding to high-potassium, the proportion of gonadotrophs responding to GnRH was reduced at Cd21 but increased at Cd35 and Cd56 (*P = 0.015, **P = 0.002). (D) Proportion of gonadotrophs exhibiting spontaneous Ca^2+^ activity before the application of GnRH and high potassium (*P = 0.011). (E) Changes in the proportion of the Ca^2+^ mobilization pattern in response to GnRH stimulation (*P = 0.047, ***P < 0.001). Kruskal–Wallis test with Dunn’s multiple comparisons and Holm correction for *P*-value adjustment; ns, non-significant. *n* = 5 for the Cd21 group, and 6 for all other groups in panels C–E.

For each animal, the ROIs were manually selected based on the intracellular Ca^2+^ response of pituitary cells to high-potassium stimulation, which was used to assess cell viability at the end of the experiment. A 10 nM GnRH stimulus was applied to identify the subpopulation of responsive cells, which we consider to be gonadotrophs, using the same set of ROIs (Fig. 3A and B). Taking the number of cells responsive to high-potassium as 100% of the cells analyzed, we identified that 27.00 ± 4.91% of the imaged cells in the control group responded to GnRH (Fig. 3C). Interestingly, in Cd21 mice, this proportion was significantly reduced to 7.44 ± 5.21%. In contrast, the Cd35 and Cd56 groups exhibited a significant increase in the proportion of GnRH-responsive cells, reaching 30.70 ± 2.47% and 39.20 ± 6.44%, respectively (see Fig. 3C).

After identifying gonadotrophs using GnRH stimulation, we investigated whether these cells exhibited basal (spontaneous) intracellular Ca^2+^ activity, a phenomenon previously associated with enhanced cellular responsiveness and secretory capacity (Van Goor *et al.* 2001). In control mice, 42.8 ± 10.4% of gonadotrophs exhibited Ca^2+^ transients. This proportion increased to 56.7 ± 8.48% in Cd21, 66 ± 20.2% in Cd35, and 77.6 ± 14.9% in the Cd56 group (Fig. 3B and D).

Furthermore, although the three principal Ca^2+^ mobilization patterns – oscillatory, biphasic, and transitory – could be identified in GnRH-challenged gonadotrophs (see the traces of individual cells from the control group in Fig. 3B), cadmium exposure altered the predominant response pattern. In control mice, the oscillatory pattern of [Ca^2+^]_i_ mobilization predominated, observed in 77.53 ± 19.64% of GnRH-responsive cells. The biphasic pattern was observed in 22.40 ± 19.6% of cells, while the transitory pattern was rare, occurring in only 0.04 ± 0.13% of cells. Importantly, cadmium exposure shifted the dominant mobilization pattern from oscillatory to biphasic (Fig. 3E). For example, in Cd21 mice, nearly all gonadotrophs responded with a biphasic pattern (99.72 ± 0.56%), with only 0.32 ± 0.53% exhibiting oscillatory activity; the transitory pattern was not observed. In Cd35 and Cd56 groups, the proportion of oscillatory responses increased to 31.40 ± 16.33% and 20.26 ± 10.82%, respectively, although the biphasic pattern remained the most frequent in both (64.67 ± 11.82% for Cd35 and 78.00 ± 8.79% for Cd56).

### Perturbation of the gonadotroph population

**Figure 4:**
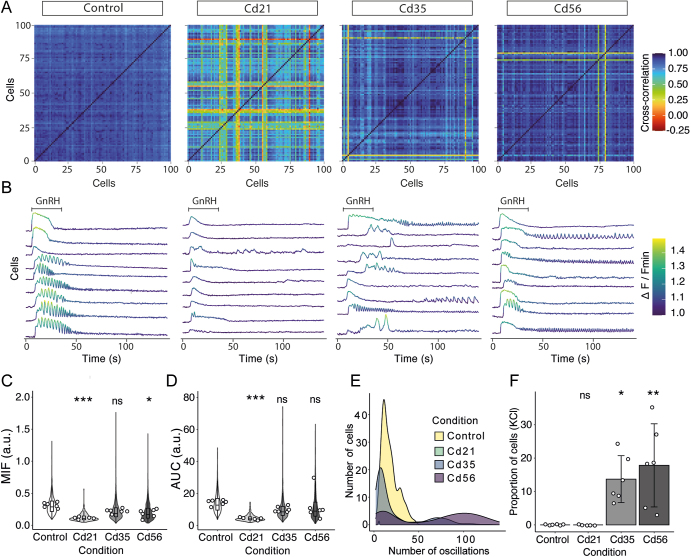
Effects of cadmium on gonadotroph population Ca^2+^ activity. (A) Representative examples (one mouse per condition) of cross-correlation analysis of GnRH-induced Ca^2+^ mobilization in gonadotrophs. (B) Ca^2+^ activity traces from individual gonadotrophs responding to 10 nM GnRH, corresponding to the examples in (A). Note the homogeneous response to GnRH in the Cd21 condition, and the long-lasting oscillations observed in Cd35 and Cd56. (C) Violin and box plots showing the maximal intensity of fluorescence (MIF) after GnRH application in gonadotrophs from control (366 ± 147 cells), Cd21 (63 ± 48 cells), Cd35 (242 ± 91 cells), and Cd56 (287 ± 167 cells) groups. Dots represent individual animals, with each dot corresponding to the mean MIF of individual cells per animal. These mean values were used for statistical analysis (*P = 0.042, ***P = 0.004). (D) Violin and box plot of the area under the curve (AUC) of Ca^2+^ mobilization following GnRH stimulation, based on the same animals and cells shown in (C) (***P < 0.001). (E) Quantification of the number of oscillations in gonadotrophs responding with the oscillatory pattern of Ca^2+^ mobilization. (F) Proportion of gonadotrophs displaying oscillations in response to high-potassium stimulation (*P = 0.011, **P = 0.008). Kruskal–Wallis test with Dunn’s multiple comparisons and Holm correction for *P*-value adjustment; ns, non-significant. *n* = 5 for the Cd21 group, and 6 for all other groups in panels C–F.

Gonadotrophs form a network of interconnected cells within the pituitary gland, allowing them to respond synchronously to stimuli and regulate gonadotropin secretion in a pulsatile manner (Fontaine *et al.* 2020). Given the significant increase in gonadotropin secretion observed in Cd35 and Cd56 mice, we hypothesized that enhanced coordination of the gonadotroph response to GnRH might contribute to this effect. To investigate this, we analyzed the gonadotroph synchrony using cross-correlation analysis of our Ca^2+^ imaging data. Although this method does not directly reveal mechanisms of cell–cell communication, it provides a quantitative measure of similarity between individual cellular responses. In control mice, most cells exhibited correlation values close to 1, indicating a uniform response to GnRH stimulation (Fig. 4A). However, these values never reached 1, likely reflecting the different patterns of Ca^2+^ mobilization observed within the population (see individual Ca^2+^ traces in Fig. 4B). In contrast, gonadotrophs from Cd21 mice markedly reduced correlation values, with some cell pairs even displaying negative correlation. Furthermore, we observed a partial recovery of response synchrony at 35 and 56 days post-treatment (Fig. 4A), suggesting that enhanced gonadotropin secretion in Cd35 and Cd56 mice is not attributable to increased synchrony of the gonadotroph response to GnRH.

We next evaluated the maximal intensity of fluorescence (MIF) and the area under the curve (AUC) of individual gonadotrophs – two parameters that quantify intracellular calcium ([Ca^2+^]ᵢ) mobilization and are positively correlated with hormone secretion (Iida *et al.* 1991). In control animals, MIF and AUC values displayed high variability across the cell population. However, this heterogeneity was markedly reduced following cadmium exposure (Fig. 4C and D). In Cd21 mice, the cellular response appeared highly homogeneous, with most cells exhibiting diminished MIF and AUC values. Specifically, MIF decreased by 60.49% relative to controls (1.31 ± 0.14 ΔF/F in controls vs 1.12 ± 0.05 ΔF/F in Cd21). Similarly, AUC values were reduced by 66.05% (4.76 ± 1.95 a.u. in Cd21 vs 14.03 ± 6.10 a.u. in controls). Partial recovery of these parameters was observed in the Cd35 and Cd56 groups, although values remained below those of controls. In Cd35 mice, MIF was reduced by 27.52% (1.23 ± 0.16 ΔF/F), and in Cd56 mice by 33.96% (1.21 ± 0.13 ΔF/F). AUC values in Cd35 were 10.32 ± 6.88 a.u., representing a 29.42% reduction, while in Cd56 the AUC decreased by 13.57% (12.13 ± 9.32 a.u.). A positive correlation was observed between MIF and AUC across all groups, indicating that cells with higher AUC values also tended to exhibit higher MIF (data not shown).

Finally, we quantified the number of calcium spikes in gonadotrophs exhibiting the oscillatory pattern of Ca^2+^ mobilization following GnRH stimulation. In control mice, the number of oscillations per cell was highly variable, with an average of 17.60 ± 9.06 spikes, ranging from 3 to 47 spikes (Fig. 4E). In Cd21 mice, oscillatory activity was nearly absent and observed only in two of the five animals analyzed. Among those cells, the average number of spikes was 5.50 ± 0.71, with a minimum of 5 and a maximum of 6. This pattern changed significantly at 35 and 56 days post-cadmium injection, showing a notable increase in the number of oscillations (23.9 ± 27.3 in Cd35 and 64.6 ± 45.6 in Cd56). The minimum number of oscillations in these groups was similar to the controls (2 in Cd35 and 3 in Cd56).

In Cd35 mice, a subpopulation of gonadotrophs exhibited irregular and erratic oscillations, with some cells displaying delayed [Ca^2+^]_i_ responses to GnRH. This may account for the reduced correlation values observed in the overall population response (Fig. 4A, B, E). In addition, we identified another distinct subpopulation in both Cd35 and Cd56 groups that exhibited prolonged oscillatory activity beginning during or shortly after GnRH stimulation, and we refer to this as long-lasting oscillatory activity (Fig. 4B). The maximum number of spikes was 126 and 140 in the Cd35 and Cd56 groups, respectively (Fig. 4B and E). Interestingly, this pattern of Ca^2+^ mobilization also appeared during high-potassium stimulation applied at the end of the recording, but only in GnRH-responsive cells exhibiting long-lasting oscillations (see Figs 3B and 4F).

Together, these findings indicate that at 21 days post-cadmium exposure, gonadotroph signaling is impaired despite cell expansion and increased *Gnrhr* expression. By days 35 and 56, the Ca^2+^ mobilization values of the population resemble those of control mice, though this recovery does not fully account for the observed increase in hormone secretion. However, the emergence of a subpopulation with long-lasting oscillations of Ca^2+^ – combined with gonadotroph expansion, enhanced basal activity, and increased GnRH responsiveness – may underlie the sustained elevation in gonadotropin secretion.

### The extracellular origin of long-lasting oscillations of Ca^2+^ mobilization

Given that the pattern of sustained Ca^2+^ oscillations was observed at 35 and 56 days – but not at 21 days – post-cadmium injection, and considering its potential association with increased gonadotropin secretion (Dupont *et al.* 2010), we next investigated whether these oscillations were driven by cadmium’s effects on either the plasma membrane oscillator or the endoplasmic reticulum oscillator (Stojilkovic & Tomic 1996). To distinguish between GnRH receptor desensitization and pharmacologically induced alterations in Ca^2+^ signaling, we first determined the minimum recovery time required for gonadotrophs to regain responsiveness following GnRH stimulation.

Using Ca^2+^ imaging, we applied two sequential pulses of 10 nM GnRH, separated by 15-, 30-, or 45-min washout periods using Ringer’s solution. We then analyzed the MIF and AUC to quantify [Ca^2+^]_i_ mobilization. When the washout interval was 15 or 30 min, gonadotrophs consistently exhibited significantly reduced MIF and AUC values in response to a second GnRH stimulus. In contrast, after a 45-min washout, gonadotrophs recovered their initial response values, indicating that this interval is sufficient to restore full responsiveness to GnRH (Supplementary Figure 2).

**Figure 5:**
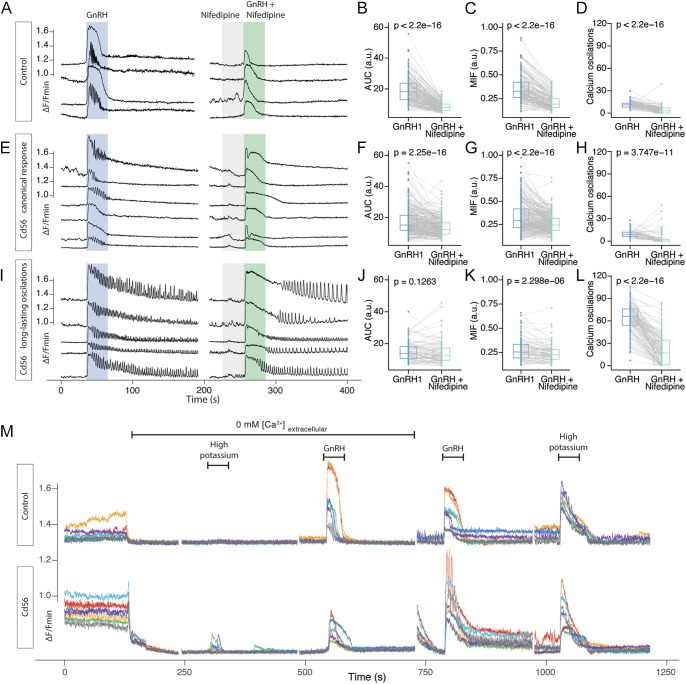
Disruption of cell membrane Ca^2+^ oscillator generates the long-lasting oscillations in gonadotrophs. (A) Four representative cells from a control mouse, before and after application of nifedipine. (B–D) Application of the L-type Ca^2+^ channel blocker nifedipine reduced Ca^2+^ mobilization, as measured by AUC, MIF, and number of oscillations; 180 cells analyzed. (E–H) In the Cd56 group, a proportion of gonadotrophs exhibited Ca^2+^ mobilization patterns similar to those in the control group, but the inhibitory effect of nifedipine was less pronounced; 280 cells from a representative mouse. (I–L) Long-lasting Ca^2+^ oscillations were reduced in magnitude but not abolished by nifedipine. Same mouse from panels E–L; 90 cells analyzed. (M) Changes in gonadotroph [Ca^2+^]_i_ activity following application of Ca^2+^-free Ringer’s solution containing EGTA. In control mice, gonadotrophs lost spontaneous activity and failed to respond to a Ca^2+^-free high-potassium stimulation. In contrast, some gonadotrophs from Cd56 mice displayed peaks of activity under the same conditions. Long-lasting oscillations were blunted but recovered when the extracellular Ca^2+^ was returned to the system. Wilcoxon signed-rank test with continuity correction.

Given that L-type Ca^2+^ channels are key components of the plasma membrane-driven Ca^2+^ oscillations in gonadotrophs (Stojilkovic *et al.* 2017), we next explored whether changes in their activity contribute to the emergence of long-lasting Ca^2+^ oscillations. To this end, we compared MIF, AUC, and number of oscillations in cells stimulated with GnRH alone or in combination with nifedipine. In control mice, nifedipine significantly reduced all Ca^2+^ mobilization parameters (Fig. 5A, B, C, D). In Cd56 mice, gonadotrophs could be grouped into two subpopulations based on their response to GnRH: one exhibited canonical patterns of Ca^2+^ activity similar to controls, while the other displayed long-lasting oscillations (Fig. 5E and I, respectively). Nifedipine reduced both MIF and AUC in these two subpopulations, although the effect was less pronounced than in control cells. Notably, the oscillations in the canonical subpopulation were significantly suppressed, whereas long-lasting oscillations were reduced in number but not entirely abolished. These results suggest that extracellular Ca^2+^ influx through L-type channels contributes to sustained oscillatory activity, but that additional intracellular mechanisms also determine this response (Fig. 5E, F, G, H, I, J, K, L).

To further investigate the role of the plasma membrane oscillator in sustained Ca^2+^ activity, we performed [Ca^2+^]_i_ measurements under extracellular Ca^2+^-free conditions. Removal of extracellular Ca^2+^ abolished spontaneous Ca^2+^ activity in gonadotrophs from both control and Cd56 mice (Fig. 5M). In addition, membrane depolarization with high potassium in a Ca^2+^-free environment failed to evoke [Ca^2+^]_i_ mobilization in control mice. In contrast, Cd56 gonadotrophs exhibited small transients resembling the transitory pattern of Ca^2+^ mobilization despite the absence of extracellular Ca^2+^ (Fig. 5M). Furthermore, both the magnitude and duration of Ca^2+^ responses – including long-lasting oscillations – were markedly reduced or eliminated in the absence of extracellular Ca^2+^, but rapidly restored upon reintroduction of extracellular Ca^2+^ into the perifusion system. In conclusion, cadmium disrupts normal plasma membrane-mediated Ca^2+^ signaling, leading to sustained gonadotroph activation that may underlie the increased gonadotropin secretion in Cd35 and Cd56 mice. However, since inhibition of the L-type Ca^2+^ channel did not fully abolish long-lasting oscillations, our data suggest that additional intracellular mechanisms contribute to this prolonged activity.

### Changes of intracellular signaling in gonadotrophs

**Figure 6:**
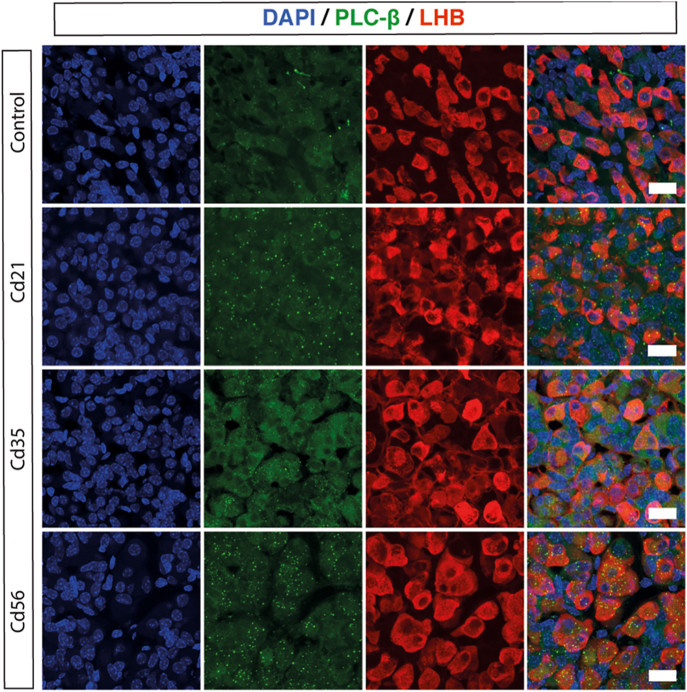
Cadmium activates intracellular signaling pathways in gonadotrophs. Representative immunofluorescence images showing the activated form of PLC-β (green) in LH-expressing gonadotrophs (red). DAPI is shown in blue. Note that PLC-β is also activated in LH-negative pituitary cells. Scale bars: 20 µm.

In addition to enhanced activity of the plasma membrane Ca^2+^ oscillator following cadmium exposure, we hypothesized that GnRH-stimulated intracellular signaling may also be disrupted, contributing to the increase of gonadotropin secretion. Specifically, we examined PLCβ, which becomes phosphorylated and activated upon GnRH receptor stimulation (Stojilkovic *et al.* 2017). Colocalization analysis of LHB and phospho-PLCβ revealed a marked upregulation of phospho-PLCβ activity in gonadotrophs from Cd21, Cd35, and Cd56 mice, while minimal phospho-PLCβ signal was detected in the control group (Fig. 6). Therefore, our results suggest that GnRH-induced PLCβ signaling is potentiated by cadmium treatment, promoting prolonged calcium signaling, increased gonadotroph-specific gene expression, and elevated gonadotropin secretion.

## Discussion

Cadmium exhibits a distinct pattern of tissue-specific retention. Unlike in the testes and blood, where cadmium levels gradually decline, its persistence in the pituitary up to 56 days post-exposure suggests impaired clearance mechanisms. This observation is consistent with previous studies indicating that cadmium can exploit endogenous metal transport systems, interfering with Ca^2+^ and zinc homeostasis and promoting preferential accumulation in endocrine tissues (Wang & Du 2013). The prolonged retention of cadmium in the pituitary likely contributes to sustained endocrine disruption, particularly in gonadotroph function, where we observed persistent hyperplasia, altered intracellular Ca^2+^ signaling, and increased gonadotropin secretion. Notably, our temporal analysis revealed that the mechanisms underlying cadmium’s disruptive effects evolve over time.

Initially, gonadotroph hyperplasia and increased GnRH receptor expression may represent a compensatory response to cadmium exposure, likely driven by the lack of negative feedback from testosterone. This is accompanied by elevated expression of *Fshb* and *Cga*. However, these molecular changes were not associated with a significant increase in gonadotropin secretion, suggesting that despite enhanced hypothalamus input, gonadotrophs exhibit a reduced capacity for coordinated response to GnRH. Supporting this, we observed a loss of synchronization within the gonadotroph cell network and a reduced proportion of GnRH-responsive cells at Cd21. Furthermore, the shift from an oscillatory to a biphasic Ca^2+^ mobilization pattern has been described as a fundamental change of pituitary secretion dynamics. The biphasic Ca^2+^ response is associated with increased LH secretion and intracellular Ca^2+^ retention, which can lead to prolonged hormone release rather than finely tuned pulses (Leong & Thorner 1991, Krsmanovic *et al.* 1992). However, gonadotrophs in the Cd21 group displayed biphasic responses with significantly reduced MIF and AUC values, resembling the uncommon transitory pattern observed in control mice. This reduction in Ca^2+^ mobilization may reflect cadmium’s known ability to block Ca^2+^ channels at the cell membrane level, as its concentration in blood is high, and interfere with intracellular signaling machinery, limiting pulsatile hormone secretion. However, the upregulation of gonadotropin subunit genes may be mediated by enhanced PLCβ activation or direct interactions of cadmium with calmodulin activity (Choong *et al.* 2014). The reduction in sperm count at this stage may therefore be attributed to both modest gonadotropin secretion and direct cadmium-induced testicular toxicity (Bhardwaj *et al.* 2021*a*,*b*).

By Cd35 and Cd56, gonadotroph physiology transitions into a state of persistent functional dysregulation, with marked increase in gonadotropin secretion. This is accompanied by the increase in gonadotroph number, *Gnrhr* expression, spontaneous Ca^2+^ activity, and responsiveness to GnRH. However, FSH secretion in Cd35 mice did not increase significantly, paralleling the reduced expression of *Fshb* and *Cga*, which may be explained by the partially restored, yet still disrupted, synchrony of gonadotroph activity and the erratic responses of individual cells to GnRH, suggesting a differential effect of cadmium on FSH and LH synthesis and release. Furthermore, while the reactivation of gonadotroph signaling suggests pituitary plasticity and a form of homeostatic adaptation, the continued dysfunction of testicular parameters – including persistently low testosterone and sperm counts – points toward a disrupted state. Despite structural recovery in the testes, the sustained elevation of LH and FSH may lead to receptor desensitization, impairing Leydig and Sertoli cell function (Habberfield *et al.* 1987, Marion *et al.* 2006). Moreover, cadmium’s lack of effect on circulating estradiol requires further study, though it is known to mimic estrogenic activity in target tissues (Johnson *et al.* 2003).

In Cd35 and Cd56 mice, hyperactivation of the PLCβ, PKC, and IP_3_ pathways suggests persistent intracellular dysregulation, likely explaining the long-lasting Ca^2+^ oscillations observed following cadmium exposure. These pathways are critical for GnRH-induced gonadotropin secretion. PLCβ activation drives IP_3_-mediated Ca^2+^ release from internal stores, while PKC modulates cell excitability, gene expression, and exocytosis. The effects of cadmium on Ca^2+^ mobilization may also involve changes in both voltage-gated Ca^2+^ channels and intracellular Ca^2+^ stores, sustaining gonadotroph activity. The long-lasting Ca^2+^ oscillations observed were of extracellular origin, indicating disruption of the membrane oscillator of gonadotrophs. Although the L-type Ca^2+^ channel does not completely explain the existence of these oscillations, other voltage-gated calcium channels insensitive to nifedipine could be expressed in gonadotrophs and participate in GnRH-induced calcium signaling (Götz *et al.* 2023). The observed shift from oscillatory to biphasic Ca^2+^ mobilization in Cd35 and Cd56 groups further supports the hypothesis that cadmium interferes with intracellular Ca^2+^ buffering, contributing to excessive gonadotropin secretion and endocrine dysfunction. Altogether, our findings show that gonadotrophs are a major target of cadmium-induced endocrine disruption, with effects that evolve in a time-dependent manner and impair reproductive capacity. While this study focused on gonadotrophs, cadmium may also affect other pituitary cell types, either directly via Ca^2+^-dependent signaling pathways or indirectly through paracrine communication with gonadotrophs – a possibility warranting further *in vivo* investigation.

Long-term exposure to cadmium at low doses has been linked with hormonal imbalances, reduced fertility, and potential transgenerational effects (Henson & Chedrese 2004, Walker & Gore 2011, Bhardwaj *et al.* 2021*b*). The broader implications of these findings are significant, as human populations are exposed to cadmium through industrial emissions, batteries, cigarette smoke, and contaminated food and water, resulting in its bioaccumulation in endocrine organs, especially the pituitary and gonads (Colborn *et al.* 1993). Cadmium also poses a serious threat to wildlife, particularly aquatic species and animals in polluted environments, where it has been linked to altered reproductive cycles and population declines (Jasrotia *et al.* 2021). Its persistence in the food chain further threatens vulnerable species, including endangered populations already at risk of reproductive failure (Vos *et al.* 2000). These findings underscore the need for stronger environmental regulations, remediation strategies, and public health interventions to reduce cadmium exposure. Finally, the present study was limited to male mouse physiology. While the hypothalamic–pituitary–gonadal axis shares key regulatory mechanisms across sexes, characteristics unique to the ovary, such as the estrous cycle and the dynamics of estrogens on the uterus and mammary gland, may influence the effects of cadmium on gonadotrophs of female animals (Johnson *et al.* 2003, Henson & Chedrese 2004). This represents an important limitation that requires further investigation. In addition, there is evidence that exposure to cadmium induces a polycystic ovary syndrome (PCOS)-like phenotype in both mice and humans, characterized by an increase in LH secretion, which may potentially affect gonadotroph signaling and secretion (Abudawood *et al.* 2021, da Costa *et al.* 2021, Bhardwaj *et al.* 2024*a*).

## Supplementary materials



## Declaration of interest

The authors declare that there is no conflict of interest that could be perceived as prejudicing the impartiality of the work reported.

## Funding

This work was supported by the Secretaría de Ciencia, Humanidades, Tecnología e Innovaciónhttps://doi.org/10.13039/501100007045 (SECIHTI, previously CONAHCYT, CBF2023-2024-727, and CONACYT-ANR 273513) and Programa de Apoyo a Proyectos de Investigación e Innovación Tecnológica (PAPIIT, UNAM, IN206624). The authors thank CONAHCYT for the support of this research through a graduate scholarship to YSA (CVU: 686018).

## Author contribution statement

YSA was responsible for conceptualization, formal analysis, investigation, methodology, project administration, writing the original draft, and writing review and editing. EHA contributed to formal analysis, investigation, methodology, and writing review and editing. DOG helped in formal analysis, investigation, methodology, and writing review and editing. OMB was responsible for formal analysis, methodology, and writing review and editing. TF contributed to conceptualization, formal analysis, funding acquisition, investigation, methodology, project administration, writing the original draft, and writing review and editing.

## Data availability

All data supporting the findings of this study are available within the article and its Supplementary materials.
