# Supplementary material for: In vivo effects of cadmium on signaling and secretion of pituitary gonadotrophs in male mice are time-dependent
Source: J Endocrinol. 2025 Oct 7;267(1):e250161. doi: 10.1530/JOE-25-0161 (PMC12508670; doi:10.1530/JOE-25-0161)
Supplement: Supplementary file 1 [file supplementary_materials.pdf]

Supporting Information for

Time-dependent effects of cadmium on gonadotroph signaling and disruption of the hypothalamus-pituitary-gonadal axis.

Yorgui Santiago-Andres[a,b,*], Elizabeth Hernández Álvarez[c], Daniel Ochoa Gutierrez[d], Ofelia Morton Bermea[c], and Tatiana Fiordelisio[a,e,*]

*Yorgui Santiago-Andres and Tatiana Fiordelisio

Email: sayorgui@ciencias.unam.mx; tfiorde@ciencias.unam.mx

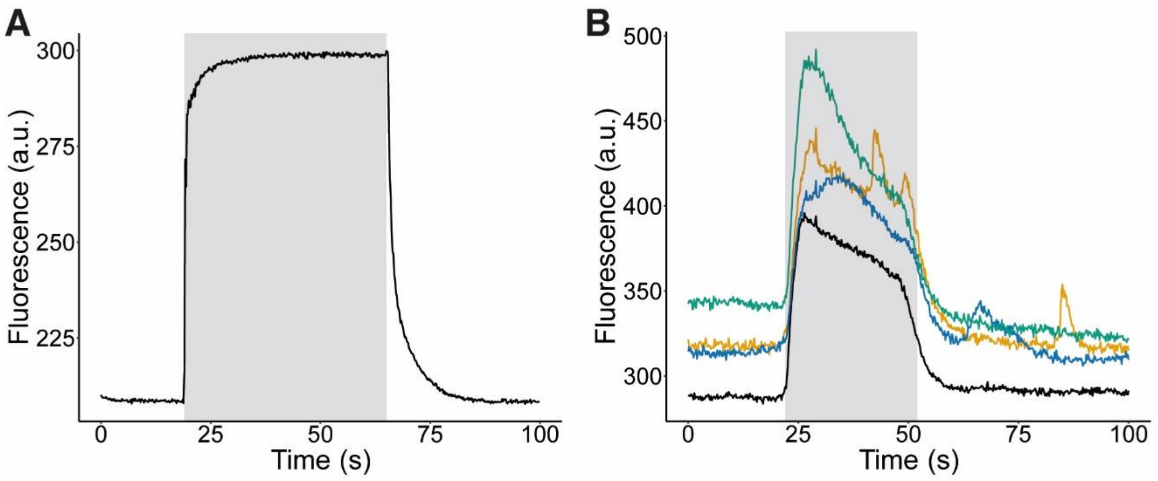

Supplementary Figure 1. Calibration of the perifusion system for Ca^{2+} imaging. (A) Perifusion of fluorescein sodium salt for 40 seconds indicated by the gray bar and showing the fast increase in fluorescence, the stable maximal fluorescence and the rapid washout of the molecule after its application. (B) Four representative pituitary cells loaded with Fluo 4-AM and stimulated for 30 sec (gray bar) with a high potassium solution. The responding cells show variation in intracellular Ca^{2+} mobilization before, during or after the stimulus.

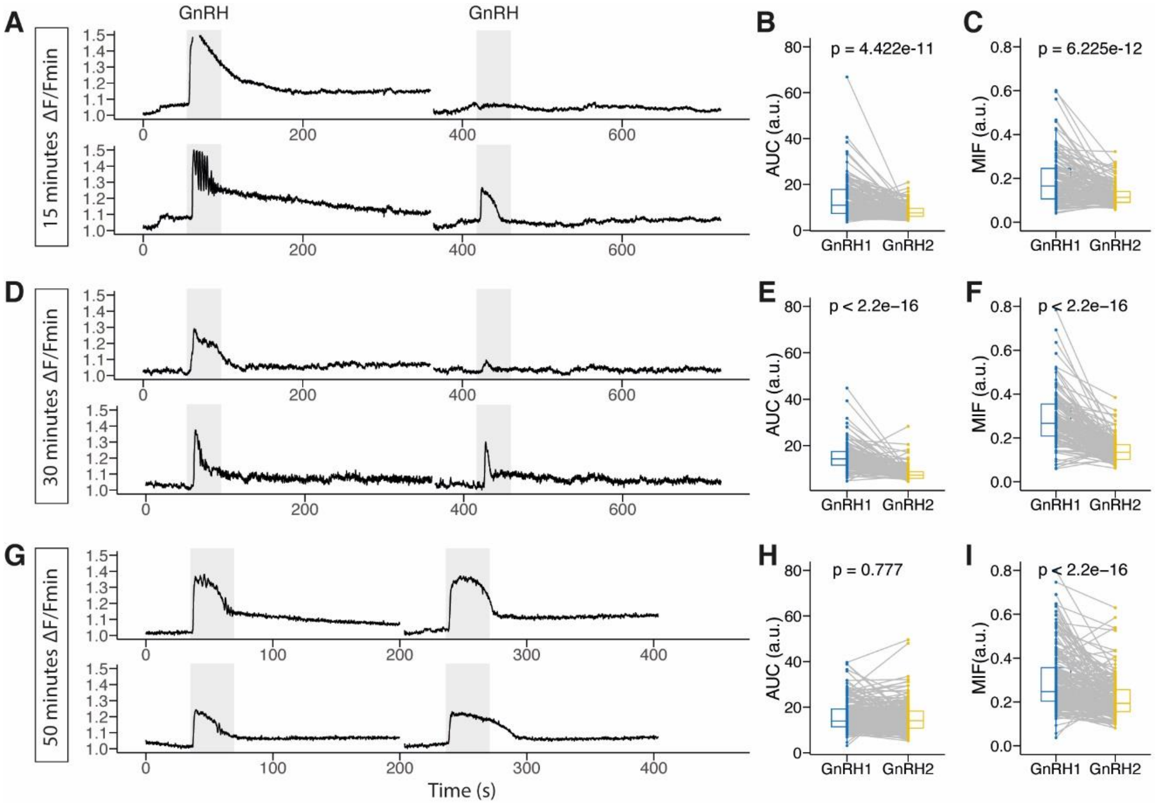

Supplementary Figure 2. GnRH receptor desensitization in gonadotrophs and analysis of recovery 15, 30 or 50 minutes after a first GnRH stimulus. (A-C) 15-minute washout with Ringer solution between two stimuli of 10 nM GnRH. (A) Two representative cells responding to GnRH applied for 30 seconds (indicated by gray bars). The breaks in the x-axis correspond to the washout period. (B and C) The second GnRH response showed lower values of AUC and MIF when compared to the first stimulus, indicating poor recovery after GnRH receptor desensitization. (D-F) 30-minute washout with ringer solution between two stimuli of 10 nM GnRH does not recover the parameters of Ca^{2+} mobilization analyzed. (G-I) 50-minute washout recovers the values of Ca^{2+} activity in the majority of the gonadotrophs analyzed. Wilcoxon signed rank test with continuity correction.
